# Epilepsy and Neurodevelopment Outcomes 24 Months after Neonatal Hypoxic–Ischemic Encephalopathy and Predictive Factors of Post-neonatal Epilepsy

**DOI:** 10.1055/a-2781-6844

**Published:** 2026-01-29

**Authors:** Graziamaria Cicala, Ornella Ricca, Maria Picilli, Elisa Rolleri, Marco Perulli, Ilaria Contaldo, Chiara Veredice, Michela Quintiliani, Maria Luigia Gambardella, Ida Turrini, Elisa Pede, Domenico Marco Romeo, Patrizia Bergonzini, Licia Lugli, Domenica Immacolata Battaglia

**Affiliations:** 1 Pediatric Neuropsychiatry, Dipartimento di Scienze della Salute della Donna, del Bambino e di Sanità Pubblica, Fondazione Policlinico Universitario Gemelli, IRCCS - Università Cattolica del Sacro Cuore, Rome, Italy; 2 Pediatric Unit, Dipartimento di Scienze Mediche e Chirurgiche Materno-Infantili e dell'adulto, Azienda Ospedaliero-Universitaria Policlinico di Modena, Modena, Italy

**Keywords:** hypoxic–ischemic encephalopathy, post-neonatal epilepsy, 24 months epileptic outcome, epileptic spasms, 24 months neurodevelopment outcome, therapeutic hypothermia

## Abstract

**Introduction:**

This retrospective, dual-center Italian study assessed the incidence, electroclinical characteristics, and risk factors for post-neonatal epilepsy among neonates with hypoxic–ischemic encephalopathy (HIE) treated with therapeutic hypothermia (TH). The study aims to better define the long-term risk factors for developing epilepsy or neurodevelopmental issues.

**Methods:**

We included neonates with HIE who underwent TH. Neurological examination and general movements were assessed before and after TH. Amplified-integrated EEGs (aEEG) or polygraphic EEGs (pEEG) were performed within 6 hours of life; a pEEG was performed after TH (72 hours to 10 days) and at 3, 9 to 12, and 24 months, and then yearly. Brain MRI was conducted within 30 days. The 24-month developmental outcome was evaluated using Griffiths Mental Development Scales. The median follow-up duration was 48 months. Epilepsy was classified according to ILAE criteria.

**Results:**

We enrolled 159 patients: 15 (9.4%) developed epilepsy. Nine (5.6%) had onset before 24 months; three of them developed infantile epileptic spasm syndrome (IESS). Seizure onset was after 24 months in 6/159 individuals (3.8%). At the last follow-up, all 15 patients had focal epilepsy. Global development was pathological in 11/15 (10/15 <2SD; 1/15 <1SD). Risk factors for post-neonatal epilepsy included: MRI lesions involving the basal ganglia and thalamus ( *p* < 0.0001), severe HIE ( *p* = 0.0008), and severe anomalies on the pEEG recorded pre-TH ( *p* = 0.0032) and post-TH ( *p* = 0.0071).

**Conclusion:**

Our study confirms that post-neonatal epilepsy is rare and generally well-controlled. MRI, HIE-3, and early pEEGs are key predictors. High-risk patients should be screened for IESS in the early months, and patients with electroclinical and neuroradiological risk factors should continue long-term neurological follow-up beyond 24 months.

## Introduction

Hypoxic–ischemic encephalopathy (HIE) describes the intricate physiological, cellular, and molecular alterations resulting from perinatal asphyxia. 1 According to the World Health Organization, perinatal asphyxia (PA) represents the 3rd most common cause of neonatal death (23%). 2 HIE accounts for approximately 1.5 per 1,000 term births in high-income countries 3 and 10 to 20 per 1,000 live births in low/middle-income countries. 4 This condition can lead to early death or a range of long-term disabilities, like cerebral palsy (CP), epilepsy, intellectual disabilities, and behavioral problems. 4 5 Importantly, HIE makes up 35 to 45% of cases of neonatal seizures. 6

Therapeutic hypothermia (TH) is the standard of care for infants with HIE, cooling the whole body to a core temperature of 33.5°C for 72 hours starting within 6 hours of birth. 7

TH has been proven to be effective in reducing the risk of CP, cognitive impairment, learning disabilities, as well as epilepsy in infants with moderate or severe HIE. 8

Infants with severe HIE seem to have a comparable seizure burden between the cooled and uncooled groups, while TH appears to have a protective effect in infants with moderate HIE. 9 Although the number of seizures is similar in the two groups and remains high in cooled infants, the seizure burden (total duration in minutes of seizures recorded per hour of polygraphic EEG [pEEG] monitoring) is reportedly lower in patients undergoing TH. 7

The incidence rate of post-neonatal epilepsy after HIE could be variable 10 : children with a history of HIE have a 5 times greater risk of developing epilepsy than controls. 11

Several studies have identified a protective effect of TH on the development of post-neonatal epilepsy. 12 13 In a retrospective study performed at the neonatology department of the University Hospital of Modena, the incidence of post-neonatal epilepsy in cooled infants was found to be 9% compared with 35% in the uncooled cohort. 12 Risk factors related to the development of post-neonatal epilepsy include the degree of encephalopathy, the presence of neonatal seizures, and profound damage involving deep gray matter and brain stem. 14

While the short-term beneficial effects are well known, we still don't know the long-term effects of TH on the incidence of post-neonatal epilepsy.

Identifying and defining potential risk factors for post-neonatal epilepsy would allow us to gain a better understanding of the mechanisms underlying post-neonatal epilepsy, identify any modifiable risk factors, and set up adequate follow-up programs.

Therefore, this study aimed to describe the risk factors of post-neonatal epilepsy in a cohort of 159 neonates affected by HIE undergoing TH, and the neurological outcome at 24 months.

## Methods

### Study Design

This two-center retrospective study was conducted at the Child Neuropsychiatry Unit of Gemelli Hospital (Rome) and the Neonatology and Pediatric Units of the University Hospital of Modena.

### Participants

We included all the newborns: (1) who were admitted (both inborn and outborn) at the two centers between September 2013 and May 2019; (2) who suffered from moderate HIE (HIE-2) and/or severe HIE (HIE-3) and underwent TH according to the recommendations of the Italian Society of Neonatology 15 ; (3) who completed at least 24 months follow-up.

According to Sarnat et Sarnat 16 and the Italian criteria for TH, 15 only newborns diagnosed with HIE-2 and HIE-3 underwent hypothermic treatment (TH).

Exclusion criteria were: (1) the death of the newborn; (2) suspension of TH for clinical adverse event due to TH; (3) the transfer to other centers, or the failure to complete the 24-month neurological follow-up.

**Fig. 1:**
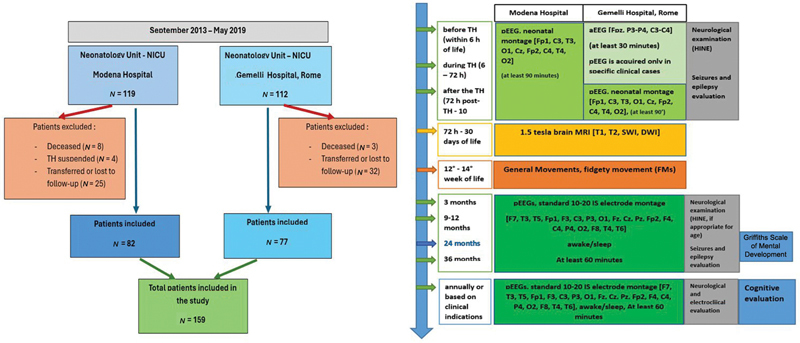
On the left inclusion and exclusion criteria for patients in our study. On the left graphical representation of clinical assessment in our cohort with the differences between the two hospitals. aEEG, amplitude-integrated EEG; pEEG, polygraphic EEG.

Finally, we included in our study 159 newborns, 82 from Modena Hospital and 77 from Gemelli Hospital. A graphical representation of our patient cohort according to the inclusion and exclusion criteria is presented in Fig. 1 .

The Ethical Committee of both hospitals approved this study.

### Assessment

Clinical data were gathered from electronic health records (EHRs) by two child neurologists and epileptologists (G.C. and O.R.).

Data regarding sex, gestational age, Apgar score at 1', 5', and 10', weight at birth, delivery mode (vaginal or C-section), intubation count, umbilical cord pH and base excess values, and the presence/absence of sentinel events (acute intrapartum events such as uterine rupture, placental abruptio, shoulder dystocia, cord prolapse, amniotic fluid embolism, sustained bradycardia in the presence of a previously normal fetal heart rate) were collected.

At Modena Hospital, neonates eligible for TH are monitored with pEEG, adapted to neonatal montage (Fp1, C3, T3, O1, Cz, Fp2, C4, T4, O2) before TH (within 6 hours of life), during TH, and after the TH (between 72 hours post-TH and 10 days).

At Gemelli Hospital, neonates eligible for TH are monitored before TH (within 6 hours of life) and during TH with an amplified-integrated EEG (aEEG) (Fpz, P3-P4, C3-C4); pEEG is acquired only in specific clinical cases; for all neonates, between 72 hours and the first 10 days of life, a pEEG (Fp1, C3, T3, O1, Cz, Fp2, C4, T4, O2) has been recorded. pEEGs were recorded for at least 90 minutes.

Two pediatric neurologists with expertise in child epileptology (G.C. and O.R.) reviewed each EEG recording. Files were reviewed using “EB Neuro Galileo” at Modena Hospital and “Micromed software” at Gemelli Hospital; pediatric neurologists were able to format montages as needed for interpretation.

We considered only pEEG recordings because pEEG is the gold standard method for assessing neonatal brain activity 17 and they were present for the majority of patients.

The alterations in neonatal pEEGs were classified according to the Murray classification: grade 0, normal EEG findings such as continuous background pattern with normal physiologic features such as anterior slow waves; grade 1, normal/mild abnormalities such as continuous background pattern with slightly abnormal activity (e.g., mild asymmetry, mild voltage depression, or poorly defined SWC); grade 2, moderate abnormalities such as discontinuous activity with interburst interval (IBI) of 10 seconds, no clear sleep–wake cycling (SWC), or clear asymmetry or asynchrony; grade 3, major abnormalities such as discontinuous activity with IBI of 10- to 60-second severe attenuation of background patterns, or no SWC; grade 4, inactive EEG findings such as background activity of 10µV or severe discontinuity with IBI of 60 seconds. 18 The presence/absence of neonatal seizures found on the EEG was assessed by reviewing the available clinical data and defined according to the recent ILAE seizure classification of the neonatal period. 19 The possible presence of neonatal status epilepticus was also evaluated according to the definition currently most accepted, which requires the NSE as continuous seizure activity for at least 30 minutes or recurrent seizures for 50% or more of a 1-hour epoch. 20 We also collected information on the type of anti-seizure medications (ASMs) received.

At Gemelli hospital, neonatal seizures were treated first with a phenobarbital loading dose; levetiracetam and phenytoin were the second and third choices. On the other hand, at Modena Hospital, the first-line treatment was phenytoin.

All patients underwent a 1.5 Tesla brain magnetic resonance imaging (MRI) (T1, T2, SWI, DWI) under normothermic conditions within the first 30 days of life. All MRI data available were classified by two expert child neurologists (G.C. and O.R.) into five patterns of brain injury as follows: pattern I, moderate/severe basal ganglia and thalami lesions associated with severe white matter damage; pattern II, basal ganglia and thalami lesions with mild or moderate white matter changes; pattern III, isolated thalamic injury; pattern IV, moderate white matter damage only; pattern V, mild white matter changes or normal finding. 21

Information relating to general movements was collected with the evaluation of spontaneous motility between the 12th and 14th week of life during the fidgety movements (FMs) period. All fidgety movements data available were reviewed and classified by two expert child neurologists (G.C. and O.R.) into: normal FMs, abnormal FMs, and absent FMs.

Following the neonatal period, neurological follow-up included both clinical and neurodevelopmental evaluations, as well as electroclinical assessments, with EEGs performed at key developmental milestones—3 months, between 9 and 12 months, 24 months, 36 months, and annually thereafter, as needed based on clinical indications. pEEGs were acquired with a standard 10–20 international system of electrode placement (F7, T3, T5, Fp1, F3, C3, P3, O1, Fz, Cz, Pz, Fp2, F4, C4, P4, O2, F8, T4, T6) with at least 1 hour of duration.

The epileptic outcome was assessed according to the International League Against Epilepsy (ILAE) criteria 22 ; the presence of epileptic syndrome at 2 years of age and after was defined according to the most recent classification. 23 24

The motor outcome was defined based on the presence/absence of CP.

The developmental outcome at 24 months of age was assessed using the Griffiths Mental Development Scales. 25

## Statistical Analysis

Data were analyzed using Medcalc software (version 8.0.0). Mean and standard deviation (SD) were calculated for the clinical characteristics of the population examined. The Chi-square test (χ ^2^ Chi-square test) was used to compare categorical variables. The Mann-Whitney test was used to compare continuous variables. To evaluate the possible associations between independent and dependent variables, we used logistic regression tests and multivariate analysis with the Stepwise model. Values of *p* < 0.05 were considered statistically significant.

## Results

Our study included 159 patients, 77 from the Gemelli Hospital in Rome and 82 from the Modena Hospital.

A description of the neonatal characteristics of the population is presented in Table 1 .

**Table 1:** Neonatal characteristics of our population: sex, gestational age, weight at birth, delivery mode (vaginal or C-section), intubation count, umbilical cord pH, umbilical cord pH base excess values, and the presence/absence of sentinel events (uterine rupture, placental abruptio, shoulder dystocia, cord prolapse, amniotic fluid embolism, sustained bradycardia in the presence of a previously normal fetal heart rate)

Population description	Total nr = 159
Gestational age (mean, SD)	39.3 ± 1.49
Weight at birth (g) (mean, SD)	3,332.9 ± 557.9
Sex (male–female) *n* , %	97 (61%) 62 (39%)
Mode of delivery ( *n* = 153) *n* , % Vaginal C-section	60 (39.2%) 93 (60.8%)
Apgar score Apgar 1' mean, DS Apgar 5' mean, DS Apgar 10' mean, DS	2.7 ± 0.74 4.9 ± 0.41 6.13 ± 0.29
Intubation count ( *n* = 151) *n* , %	113 (74.8%)
Umbilical cord pH (mean, SD)	6.96 ± 0.19
Umbilical cord pH base excess values (mean, SD)	17.34 ± 5.62
Sentinel event ( *n* = 131) *n* %	53 (40.5%)

Abbreviation: SD, standard deviation.

### Encephalopathy Grade

Given the retrospective nature of the study, it was not possible to determine the encephalopathy grade for all patients. Among the remaining cases (113/159), 89% exhibited HIE classified as moderate or severe within the first 6 hours of life: HIE-2, moderate, in 63.8% ( *n* = 81); HIE-3, severe, in 25.2% ( *n* = 32). Moreover, we found severe (grade 3) encephalopathy in 91% ( *n* = 10/15) of infants with a subsequent diagnosis of post-neonatal epilepsy and in 12% of non-epileptic patients.

### EEG Findings

A total of 53% patients had a pEEG recording performed in the first 6 hours of life. Most of the recordings documented moderate (grade 2 in 50.6%) and severe (grade 3 in 44.7%) anomalies; only 4.7% had inactive traces (grade 4), and there were no cases of mild anomalies (grade 1). In the neonatal intensive care unit (NICU), 61 patients (38.4%) experienced electrographic-only seizures, and 8 patients presented a status epilepticus. A post-hypothermic pEEG between 72 hours and 10 days of life was available for 97 patients (61%). Most of the pEEGs were normal or showed slight anomalies (84.5%): in particular, normal traces were found in 42.2% of cases (40/97) and grade 1 anomalies in 43.3% of the traces analyzed (42/97). Of the remaining 15 recordings, 10 documented moderate anomalies (10.3%), and 5 documented severe anomalies (5.2%). During the hospitalization in the NICU, 72 patients (45.3%) underwent ASMs. The most used ASMs were phenobarbital (50/72 patients; 69.4%) and phenytoin (30/72 patients; 41.7%).

Finally, 93.3% of infants with post-neonatal epilepsy had neonatal seizures during their stay in the NICU, which was significantly higher than in the non-epileptic group (33.6%).

### MRI Findings

All patients underwent brain MRI within 1 month of life (median age 8.2 days of life). In 48 patients damage to the basal ganglia and thalamus was seen (pattern 1–2-3): 18 (11.3%) pattern I, 18 (11.3%) pattern II, and 12 (7.5%) pattern III.

### General Movements

The evaluation of general movements was performed in 131/159 patients. In 24 children, fidgety movements were absent, and in 9 children, fidgety movements were abnormal. In all subjects with CP for which data were available, fidgety movements were abnormal or absent.

### Motor Outcome

Neurological follow-up at 24 months ascertained that 13 of the 159 infants had developed CP. Early brain MRI of 12 out of 13 infants with CP documented severe basal ganglia and thalamic damage.

### Cognitive Outcome

We evaluated the developmental outcome using the Griffiths Mental Development Scales (GMDS-II) at 24 months of age. The total developmental quotient (DQ) was 2 standard deviations (SD) below the norm in 13 patients (8.2%). In 83.6% of patients, the 24-month DQ was normal. In 11/13 (84.6%) infants with pathological DQ, MRIs showed lesions of the basal ganglia and thalami, which were significantly higher than those with normal DQ of 25.5% (37/145). The Griffiths subscale with higher pathological values was the language: 34 patients scored <2 SD.

Among the 15 epileptic patients, the DQ was normal in 4 of them, and in 11/15 it was pathological (10/15, <2 DS; 1/15, <1 DS).

#### Language Outcome

Among 34 patients without CP or a below-average global DQ, 11 still obtained an abnormal score in the language area of the GMDS-II evaluation; none of them presented epilepsy. Out of 11 patients 3 had a pattern of severe damage involving the basal ganglia and thalamus (pattern I). Their pEEGs evidenced: in 5/11 mild to moderate epileptic anomalies (recordings made within the first 20 days of life), in 1/11 focal anomalies in wakefulness (recording made between 2 and 6 months of life), 1/11 had focal epileptic anomalies in wakefulness at the 36-month recording, and epileptic anomalies in wakefulness that were intensified during sleep at the recording made at 4 years.

### Epilepsy Outcome

Patients with post-neonatal epilepsy were 15/159 (9.4%): 9/159 (5.6%) with onset before 24 months (range 1–21 months), 7 with onset before 6 months of life. Moreover, 6/159 (3.8%) patients had epilepsy onset after 24 months of age (median age of onset: 33 months, range between 27 and 48 months).

At seizure onset, 3/15 patients had infantile epileptic spasms syndrome (IESS) between 5 and 6 months of age: only two-thirds presented clinical spasms and one-third was diagnosed thanks to a screening EEG. All the other patients presented focal seizures at their onset.

The median duration of follow-up for all patients was 48 months. Out of 15 patients 10 currently have seizures: 3/10 with monthly frequency, 7/10 with annual frequency, and only one patient has no seizures after 24 months of age.

Status epilepticus occurred in two infants. One of them presented a focal status epilepticus when he was 3 years old; unluckily, we have no more specific information about the other one.

Out of 15 patients 13 are currently on ASMs, 5 of whom are in monotherapy. Patients (9/15) with the onset of epilepsy within 2 years of age showed a significantly ( *p* = 0.0052) higher incidence of CP and delayed psychomotor development (100%) than those with the onset of epilepsy at >24 months (16.7%) (see Table 2 ).

**Table 2:** Patients with epilepsy at the last follow-up

ID	Prognostic factors							Motor and developmental outcome			Epileptic outcome						
Patients	Sex	HIE	6 hours of life EEG	Post TH EEG	CN	MRI	ASMs in neonatal period	Fidgety	CP	DQ	Type of seizures at onset	Age at seizure onset (months)	Follow-up duration (months)	Seizures (last year of follow-up)	Seizures' frequency (last year of follow-up)	Current ASMs	Current epilepsy
1	F	3	3	3	Yes	I	PB, PHT, MDZ	Abn	TP	<2 DS	F	48	96	Yes	Yearly	VPA, LVT	Focal
2	M	3	3	2	Yes	II	PB, PHT, MDZ	Abs	TP	<2 DS	F	27	120	No	/	LVT, PB	Focal
3	F	3	3	–	Yes	I	PHT, MDZ	N	/	<1 DS	F	36	84	Yes	Yearly	LVT	Focal
4	M	3	3	–	Yes	I	PB, MDZ, LVT	Abs	TP	<2 DS	F	5	60	No	/	LVT	Focal
5	F	3	3	–	Yes	I	PHT, MDZ	Abs	TP	<2 DS	F	1	38	Yes	Yearly	LVT, PB, TPM	Focal
6	M	3	4	3	Yes	I	PHT, MDZ, LVT	Abs	TP	<2 DS	F	4	30	Yes	Yearly	VPA, LVT, CLZ	Focal
7	M	3	4	0	Yes	III	PHT, MDZ	Abs	TP	<2 DS	F	21	32	No	/	LVT	Focal
8	F	–	–	–	Yes	IV	PB	Abn	/	Normal	F	36	96	No	/	LVT	Focal
9	M	–	–	1	No	V	–	N	/	Normal	F	28	60	Yes	Yearly	None	Self-limited epilepsy with autonomic features
10	M	3	–	1	Yes	III	PB	N	/	Normal	F	30	48	No	/	None	Focal
11	M	–	–	–	Yes	I	PB, PHT, LVT	–	TP	<2 DS	F	18	48	Yes	Yearly	VPA, LVT	Focal
12	M	–	–	0	Yes	V	PB	–	/	Normal	F	2	7	Yes	Monthly	VPA	Focal
13	F	3	–	–	Yes	I	PB, MDZ, LVT	Abs	TP	<2 DS	IESS	5	48	Yes	Yearly	VPA, LVT	Focal
14	M	3	–	3	Yes	I	PB, MDZ	–	TP	<2 DS	IESS	5	48	Yes	Yearly	VPA, CLZ	Focal
15	F	2	3	–	Yes	I	PB, LVT	Abs	TP	<2 DS	IESS	6	42	Yes	Yearly	LVT	Focal

Abbreviations: Abn, abnormal; Abs, absent; CLZ, clonazepam; CP, cerebral palsy; DQ, developmental quotient; F, focal seizure; HIE, hypoxic–ischemic encephalopathy; IESS, infantile epileptic spasms syndrome; LVT, levetiracetam; MDZ, midazolam; N, normal; PB, phenobarbital; PHT, phenytoin; TP, tetraparesis; TPM, topiramate; VPA, sodium valproate.

Notes: EEG patterns: grade 0, normal EEG findings; grade 1, normal/mild abnormalities; grade 2, moderate abnormalities; grade 3, major abnormalities; grade 4, inactive EEG findings. 18

MRI patterns: pattern I, basal ganglia and thalami lesions associated with severe white matter damage; pattern II, basal ganglia and thalami lesions with mild or moderate white matter changes; pattern III, isolated thalamic injury; pattern IV, moderate white matter damage only; pattern V, mild white matter changes or normal finding. 21

### Combined Evaluation of the Previous Outcomes

By evaluating the different outcomes analyzed, we found that 10 patients are affected by all three conditions (CP, epilepsy, psychomotor developmental delay), 10 patients by CP and developmental delay, 5 patients by epilepsy alone, 2 patients by CP alone, and 3 patients by developmental delay alone.

### Predictive Factors

A comparison was performed to assess the differences between infants with and without epilepsy or an epileptic syndrome with onset after the neonatal period. No correlation was found with neonatal/perinatal characteristics such as gender, gestational age, birth weight, sentinel events, mode of delivery, cord values of BE, pH, and Apgar score, and the risk of epilepsy.

Univariate analysis showed a significant association between the most severe degree of encephalopathy and the development of post-neonatal epilepsy ( *p* = 0.0008). There was also a correlation between post-neonatal epilepsy and the severity of the abnormalities documented by the pEEG recordings at 6 hours ( *p* = 0.0032) and post-hypothermia (0.0071).

Pre-hypothermic pEEG at 6 hours of life was available in 8/15 patients diagnosed with post-neonatal epilepsy and 77/144 non-epileptic patients.

Comparing the pEEGs from the two groups revealed a significant difference in the prevalence of the observed anomaly grades ( *p* = 0.0009). Specifically, all EEGs from the patients with epilepsy showed severe abnormalities or an inactive tracing (grade 3: 75% of cases; grade 4: 25% of cases), whereas those from the non-epileptic patients predominantly exhibited moderate abnormalities (grade 2: 55.8%; grade 3: 41.6%; grade 4: 2.6%).

Post-TH pEEG (between 72 hours and 10 days) was performed in 8/15 epileptic and 89/144 non-epileptic patients. The comparison between the pEEGs from the two groups revealed a significant difference in the prevalence of anomaly grades ( *p* = 0.0061). EEGs from the patients with epilepsy demonstrated a persistence of moderate-to-severe anomalies (grade 2: 12.5% of cases; grade 3: 37.5% of cases), whereas those from the non-epileptic patients were predominantly normal or exhibited mild anomalies (grade 0: 42.7%; grade 1: 44.9%; grade 2: 10.1%; grade 3: 2.2%).

The presence of neonatal seizures is, in turn, related to the diagnosis of post-neonatal epilepsy ( *p* = 0.0014). We also found that the absence of damage to the basal ganglia and thalamus on MRI is a protective factor for the development of post-neonatal epilepsy ( *p* ≤ 0.0001).

In the multivariate analysis, we found that the only variable related to the development of post-neonatal epilepsy is the degree of severity of the pEEG at 6 hours according to Murray ( *p* = 0.0107).

Finally, we conducted a further multivariate analysis excluding the data of the pEEG at 6 hours and the pEEG after hypothermia, since these are not present for some of the patients. A significant correlation between the development of post-neonatal epilepsy and the severity of HIE ( *p* = 0.0128) and the magnetic resonance pattern ( *p* = 0.0102) was observed. In particular, involvement of the basal ganglia and thalamus (patterns I and II: 67.7% of cases) is more commonly found in infants with epilepsy, while white matter injuries are more frequently observed in non-epileptic infants (patterns IV and V: 74.8% of cases) ( *p* < 0.0001).

Univariate and multivariate analyses are shown in Table 3 ; the data distinguishing patients with and without post-neonatal epilepsy are shown in Table 4 .

**Table 3:** Logistic regression with evaluation of risk factors for the development of postnatal epilepsy in subjects with HIE undergoing therapeutic hypothermia

	Univariate analysis			Multivariate analysis (with pEEG pre and post TH)			Multivariate analysis (without pEEG pre and post TH)		
	OR	CI	P	OR	CI	P	OR	CI	P
Apgar score 1'	0.9018	0.6759–1.2031	0.4822						
Apgar score 5'	0.8608	0.6567–1.1282	0.2775						
Apgar score 10'	0.9572	0.7046–1.3002	0.7794						
Umbilical cord pH	0.0501	0.0020–1.2393	0.0674						
Umbilical cord BE value	1.0684	0.9493–1.2024	0.2727						
Weight at birth	0.9990	0,9980–1.0001	0.0816						
Gestational age	1.1900	0.8062–1.7565	0.3813						
Sex	0.8311	0.2988–2.3114	0.7205						
Delivery mode	1.1859	0.4345–3.2370	0.7392						
Sentinel event	1.8986	0.9550–3.7747	0.0675						
**HIE-3**	36.7394	4.5100–299.2854	**0.0008** a				17.2803	1.8339–162.8270	**0.0128** a
**Neonatal seizures**	28.8936	3.6882 to 226.3541	**0.0014** a						
**pEEG at 6 h of life**	11.6962	2.2752 to 60.1254	**0.0032** a	22.1130	2.0515–238.3592	0.0107			
**pEEG post TH (72 h and 10 days)**	2.9989	1.3490 to 6.6669	**0.0071** a						
**MRI**	0.4111	0.2721 to 0.6212	**<0.0001** a				0.3153	0.1306 to 0.7610	**0.0102** a
**GMs (Fidgety)**	3.5060	1.7662 to 6.9595	**0.0003** a						

Abbreviations: CI, confidence interval; OR, odd ratio; P, *p* -value.

Note: In the last main column results from multivariate analysis by excluding pEEGs pre-TH (at 6 hours of life) and post TH (between 72 hours and 10 days) are shown.

a values of *p* < 0.05 are considered statistically significant.

**Table 4:** Raw data, percentages, and *p* -value are displayed for patients with and without post-neonatal epilepsy

	Patients with post-neonatal epilepsy ( *N* = 15)	Patients without post-neonatal epilepsy ( *N* = 144)	P
**Encephalopathy grade**			<0.0001 a
Mild	/	/	
Moderate	1 (9%)	80 (78%)	
Severe	10 (91%)	22 (22%)	
*Not available*	4	42	
**Neonatal seizures**			<0.0001 a
Y	14 (93.3%)	47 (33.6%)	
N	1 (6.7%)	97 (67.4%)	
**pEEG at 6 h of life pre-TH**			0.0009 a
Grade 0	/	/	
Grade 1	/	/	
Grade 2	/	43 (55.8%)	
Grade 3	6 (75%)	32 (41.6%)	
Grade 4	2 (25%)	2 (2.6%)	
*Not available*	7	67	
**pEEG between 72 h and 10 days of life post-TH**			0.0061 a
Grade 0	2 (25%)	38 (42.7%)	
Grade 1	2 (25%)	40 (44.9%)	
Grade 2	1 (12.5%)	9 (10.1%)	
Grade 3	3 (37.5%)	2 (2.2%)	
Grade 4	/	/	
*Not available*	7	55	
**MRI pattern**			<0.0001 a
1	9 (60%)	9 (6.3%)	
2	1 (6.7%)	17 (11.9%)	
3	2 (13.3%)	10 (6.9%)	
4	1 (6.7%)	24 (16.8%)	
5	2 (13.3%)	83 (58%)	
*Not available*	/	1	

Abbreviations: N, not; Y, yes; /, none.

a values of *p* < 0.05 are considered statistically significant.

## Discussion

Our dual-center study showed a low frequency of post-neonatal epilepsy (9.4%) among children with HIE treated with TH. We could display a medium-term follow-up for all patients (24 months), and an even longer one for patients with epilepsy (median 48 months). Onset before 24 months occurred in 5.6% patients, and after 24 months in 3.8% patients.

Nyman and colleagues also observed a similar frequency of post-neonatal epilepsy (8.7%). 26 Their cohort had 4-year follow-up and all children had epilepsy onset before 1 year of age. 26 In another regional cohort of 14/134 patients with post-neonatal epilepsy, 27 only 8 (6%) presented seizures before 2 years, whereas the other 6 (4.5%) presented epilepsy onset between 4 and 8 years of age. 27 Therefore, they emphasized the importance of a longer follow-up, even though their follow-up duration was not uniform. 27 The strength of our study is certainly the fact that epileptic patients have a median follow-up of 48 months up to 120 months, which has also allowed us to monitor and identify those patients who develop epilepsy after 24 months.

Extended follow-up may not be feasible in every hospital or outpatient clinic, so it is crucial to identify patients at risk of developing post-neonatal epilepsy.

Nowadays, few studies have evaluated the frequency of post-neonatal epilepsy and the correlation between perinatal factors and the development of post-neonatal epilepsy with different follow-up durations. 10 26 28

In our analysis of the electroclinical features and risk factors, encephalopathy was found to be significantly more severe in patients with post-neonatal epilepsy. Severe encephalopathy is a known risk factor for the development of post-neonatal epilepsy and, more broadly, for poorer neurodevelopmental outcomes. 28 29 Van Kooij et al 10 reported that 8/80 children with mild or moderate neonatal encephalopathy developed post-neonatal epilepsy (10%), whereas Toet and colleagues observed that 6/84 (7%) patients developed post-neonatal epilepsy after moderate HIE. 30

Consistent with the literature, 31 32 our study children who developed epilepsy had a more frequent history of neonatal seizures than children who survived without epilepsy. On the other hand, as previously observed, 31 33 infants with MRI of severe damage involving the basal ganglia and the thalami are at greater risk of developing post-neonatal epilepsy. We also observed that non-epileptic infants reported damage mainly to the white matter or minimal brain involvement, and those without basal nuclei and thalami lesions have a lower risk of developing post-neonatal epilepsy than newborns with central gray matter damage. The underlying mechanism could be related to the synaptic reorganization following neuronal damage 34 of particularly hyperexcitable neuronal networks between the cortex and brainstem, related to the loss of modulation by the thalamus and basal ganglia. 35 This mechanism can lead to unprovoked and recurrent epileptic seizures, generally after a seizure-free latency period, which may, however, be absent in the most severe cases. 36

Our study enhances the importance of early pEEG recordings as a fundamental prognostic tool for assessing the epileptic outcome. We observed that more severe EEG anomalies recorded either at 6 hours or at the end of TH (72 hours to 10 days of life) correlate significantly with the development of post-neonatal epilepsy. In particular, all pEEGs performed at 6 hours on infants who developed epilepsy documented severe anomalies/inactive tracings, while the EEGs of patients without epilepsy documented predominantly moderate abnormalities. Moderate/severe abnormalities persisted in the EEGs of children with epilepsy performed between 72 hours and 10 days, unlike those of non-epileptic patients, which were predominantly normal or mildly abnormal. Nash and colleagues found that the greatest prognostic value of EEG background for predicting moderate to severe brain MRI injury was not achieved until midcooling (24–30 hours), highlighting the importance of continuous monitoring or sequential EEGs. 37 Moreover, Nyman et al demonstrated that, at the individual level, the most reliable predictors of subsequent epilepsy were an inactive aEEG at 24 hours. 26

In summary, our study suggests that in hospitals with limited resources, a pEEG at 6 hours and again at 72 hours (after TH) of life can be prioritized in place of continuous EEG monitoring.

Regarding the neurological outcome, our data indicate that patients with epilepsy with MRI damage grades 1 to 3 all have an onset within the first 4 years of life, and very often within the first 2 years with focal seizures. Notably, a patient with normal MRI and onset at 28 months of age was diagnosed with self-limited epilepsy with autonomic features. This opens the question of whether a subtle white matter damage went unnoticed, as suggested by the frequent presentation of atypical self-limited epilepsy after HIE 27 or whether this common form of epilepsy was a coincidence unrelated to HIE.

Nonetheless, the principal epileptic syndrome identified among our patients was IESS in 3/15 patients (median onset of 5 months). Structural acquired etiologies and in particular HIE are already described as the main cause of IESS and with an onset between 3 and 12 months in the majority of the cases. 38 The risk of infantile spasms is described as higher in infants with severe HIE. 13

This study has several limitations. Being retrospective and based on data from the hospital's EHRs, it is inherently subject to selection biases and to a lack of clinical data. Second, part of the discrepancy in the sample is due to the presence of patients from two different hospitals with different clinical protocols. At the same time, in real-life clinical practice, this study could be a cue to allocate resources to the most at-risk patients and focus on more targeted screening, such as that for infantile spasms in the first 12 months of life.

To conclude, our study showed how patients undergoing TH generally have a low probability of developing epilepsy, but approximately 4% have early epilepsy onset (before or at 6 months), in half of the cases with epileptic spasms. Therefore, it is necessary to raise awareness among families of at-risk patients and to perform early EEG screenings, considering that the onset of spasms is usual in epileptic spasms syndrome. 39

## Conclusion

In an unselected cohort of patients with HIE undergoing hypothermia, we found a 60% incidence of epilepsy with onset before 24 months and a 40% incidence of epilepsy with onset after 24 months of age, with a median age of onset of 33 months.

MRI showing damage involving basal ganglia and thalami, and severe grade of encephalopathy on early pEEG, at 6 and after 72 hours of life, are the main predictor of post-neonatal epilepsy. We, therefore, observed that in patients with these risk factors, it could be of help to perform very early pEEG screening and educate parents to recognize the onset of focal epilepsy or epileptic spasms. At the same time, clinical neurological and epileptic follow-up should be continued after 24 months if the risk factors already mentioned are present.
